# Lovastatin induced Kruppel like factor 2 (*KLF2*), Kruppel like factor 6 (*KLF6*) and Ras homolog family member B (*RHOB*) genes and preferentially led to viability reduction of Cisplatin-resistant cells

**DOI:** 10.18632/oncotarget.22472

**Published:** 2017-11-16

**Authors:** Chiho Koi, Hiroto Izumi, Tomoko Kurita, Thuy Thi Nguyen, Midori Murakami, Yukiko Yoshiura, Toru Hachisuga, Yasuo Morimoto

**Affiliations:** ^1^ Department of Obstetrics and Gynecology, School of Medicine, University of Occupational and Environmental Health, Kitakyushu, Fukuoka 805-8555, Japan; ^2^ Department of Occupational Pneumology, Institute of Industrial Ecological Sciences, University of Occupational and Environmental Health, Kitakyushu, Fukuoka 805-8555, Japan

**Keywords:** statin, cisplatin resistance, KLF, RHOB, HMG-CoA

## Abstract

It was reported that statins, inhibitors of 3-hydroxy-3-methylglutaryl coenzyme A reductase that are used to prevent hypercholesterolemia, have antitumor activity in several cancers. In this study, we investigated the cell viability of statins in Cisplatin-resistant HCP4 and PCDP5 cells compared with their parent Hela and PC3 cells, respectively, and found that HCP4 and PCDP5 cells were 37-fold and 18-fold more resistant to Cisplatin but 13-fold and 7-fold more sensitive to Lovastatin by cell proliferation assay. Lovastatin induced the apoptosis of HCP4 cells more rapidly and to greater extent than in Hela cells as assessed by flow cytometry and western blotting analyses. The MVA pathway was not involved in this acquired Cisplatin resistance. To elucidate the mechanism underlying the reduced viability to Lovastatin, we performed cDNA microarray analysis and identified 65 and 54 genes that were induced more than 2-fold by Lovastatin in HCP4 and PCDP5 cells, respectively. Of these, only three genes, *KLF2*, *KLF6*, and *RHOB*, were commonly induced between HCP4 and PCDP5 cells. These mRNAs were strongly induced by Lovastatin with transcriptional regulation in HCP4 cells. Consistent with transcription, the protein expression of *RHOB* also was induced by Lovastatin. The induction of these genes was associated with cell cycle arrest and apoptosis. Combination treatment with Cisplatin and Lovastatin resulted in an agonistic effect in Hela and PC3 cells and an antagonistic effect in HCP4 and PCDP5 cells. These results suggest that statins might have the potential to overcome Cisplatin resistance as single-agent therapy.

## INTRODUCTION

Gynecologic cancers, including cervical, endometrial, and ovarian cancer, constitute one of the main causes of death from cancer worldwide [1]. Patients with gynecologic cancers are often treated with combination platinum and taxane-based chemotherapy, but many patients develop recurrent disease with poor survival as a result of acquired or intrinsic drug resistance [2–4]. In particular, Cisplatin resistance is a critical issue that must be overcome when considering the treatment of gynecologic cancers, as well as several other cancers that display Cisplatin resistance.

Statins are well-known agents that inhibit HMGCR and are commonly used to prevent hypercholesterolemia [5]. Recently, statins have received increasing attention because of emerging evidence of their antitumor effects [6, 7]. Furthermore, the importance of MVA pathway metabolites and enzymes in the survival of cancer cells has been highlighted by the latest studies [8, 9].

KLFs are a subfamily of the zinc-finger class of DNA-binding transcriptional regulators and are involved in the regulation of differentiation, development, cellular proliferation, growth-related signal transduction, and apoptosis [10, 11]. It was reported that *KLF2* and *KLF6* act as tumor suppressor genes, and downregulation of *KLF2* or *KLF6* was associated with poor survival in several cancers [12–17]. *RHOB* is one of the Rho family of small GTPases, signaling molecules that regulate many cellular processes including cytoskeletal dynamics, cell motility, cell adhesion, cell division, and transcription [18]. The Rho GTPases thereby contribute to wound healing, inflammation, and cancer progression [18]. *RHOB* is also known as a tumor suppressor that promotes growth inhibition and induces apoptosis in cancer cells [19, 20].

In this study we found that statins preferentially led to viability reduction of Cisplatin-resistant cells compared with Cisplatin-sensitive cells, and that expression of *KLF2*, *KLF6*, and *RHOB* was induced in response to Lovastatin. We investigated the involvement of these tumor suppressor genes and MVA pathway-associated genes in Cisplatin resistance.

## RESULTS

### Lovastatin sensitized Cisplatin-resistant cells

**Figure 1:**
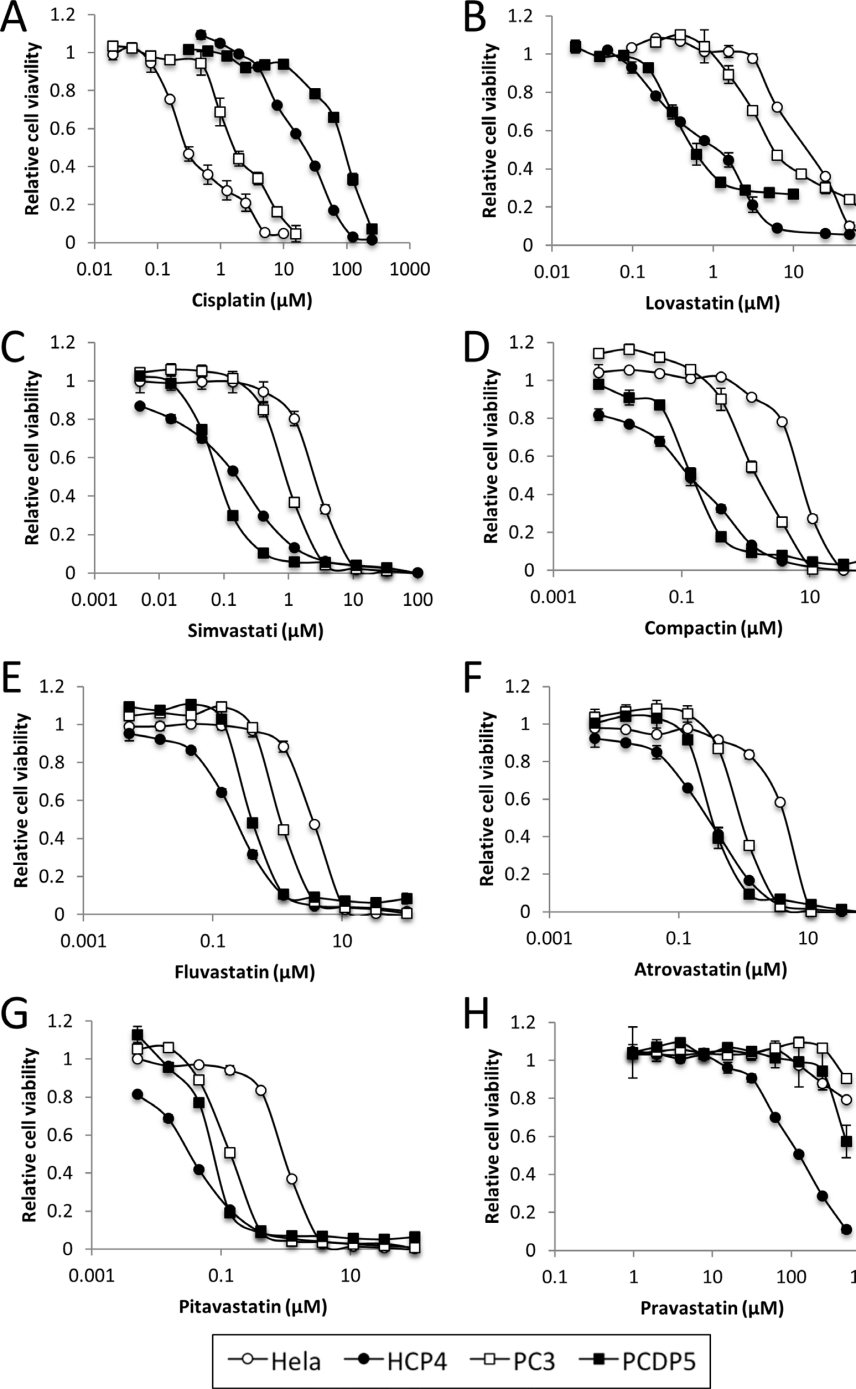
Statins sensitized Cisplatin-resistant cells Hela, HCP4, PC3 and PCDP5 cells were treated with serial dilutions of Cisplatin or seven kinds of statin. After 72 h, the surviving cells were stained with TetraColor ONE for 2–3 h. All values represent the mean of at least two independent experiments.

We evaluated the effects of Cisplatin and Lovastatin on cell viability of Cisplatin-resistant HCP4, PCDP5 cells and parental Hela, PC3 cells, respectively, by cell proliferation assay. The IC50 of Cisplatin and statins for Hela, HCP4, PC3 and PCDP5 cells were calculated with CalcuSyn software. HCP4 and PCDP5 cells were 37-fold and 18-fold more resistant to Cisplatin than their parental cells, respectively (Figure 1 and Table 1). In contrast, HCP4 and PCDP5 cells were 13-fold and 7-fold more sensitive to Lovastatin than their parental cells, respectively (Figure 1 and Table 2). HCP4 and PCDP5 cells were also more sensitive than their parental cells to other statin-related agents, including Simvastatin, Pravastatin, Compactin, Fluvastatin, Atorvastatin, Pitavastatin, and Pravastatin (Figure 1 and Table 2). We also evaluated the effects of Lovastatin on Cisplatin-resistant DDP10 cells, oxaliplatin-resistant OX2 cells and Mithramycin-resistant MM4 cells derived from T24 cells (Supplementary Table 1). DDP10, OX2 and MM4 cells were 7.1-fold, 15.6-fold and >270-fold more resistant to Cisplatin, Oxaliplatin and Mithramycin, respectively, when compared with parental T24 cells. DDP10 and OX2 cells were 1.3-fold and 2.2-fold more sensitive to Lovastatin, respectively, while MM4 cells were not sensitive to this compound.

**Table 1:** Evaluation of IC50

	Hela	HCP4	Ratio (HCP4/Hela)	PC3	PCDP5	Ratio (PCDP5/PC3)
Cisplatin (μM)	0.530 ±0.042	19.564 ±1.271	36.884	2.877 ±0.209	53.139 ±0.673	18.468

**Table 2:** Evaluation of IC50

	Hela	HCP4	Ratio (Hela/HCP4)	PC3	PCDP5	Ratio (PC3/PCDP5)
Lovastatin (μM)	12.499 ±0.859	0.927 ±0.019	13.48	10.719 ±0.131	1.508 ±0.078	7.11
Simvastatin (μM)	1.961 ±0.958	0.111 ±0.007	17.67	1.052 ±0.147	0.216 ±0.098	4.87
Compactin (μM)	5.671 ±1.589	0.083 ±0.012	68.33	1.382 ±0.136	0.291 ±0.046	4.75
Fluvastatin (μM)	2.486 ±0.943	0.245 ±0.056	10.15	2.450 ±1.120	0.878 ±1.227	2.79
Atorvastatin (μM)	1.815 ±0.069	0.188 ±0.021	9.65	0.916 ±0.046	0.593 ±0.201	1.54
Pitavastatin (μM)	0.662 ±0.065	0.028 ±0.001	23.64	0.106 ±0.008	0.104 ±0.032	1.02
Pravastatin (μM)	>500	128.637 ±12.731	N.C.	>500	>500	N.C.

N.C. indicates “not calculated”

### Lovastatin induced apoptosis of Cisplatin-resistant cells

**Figure 2:**
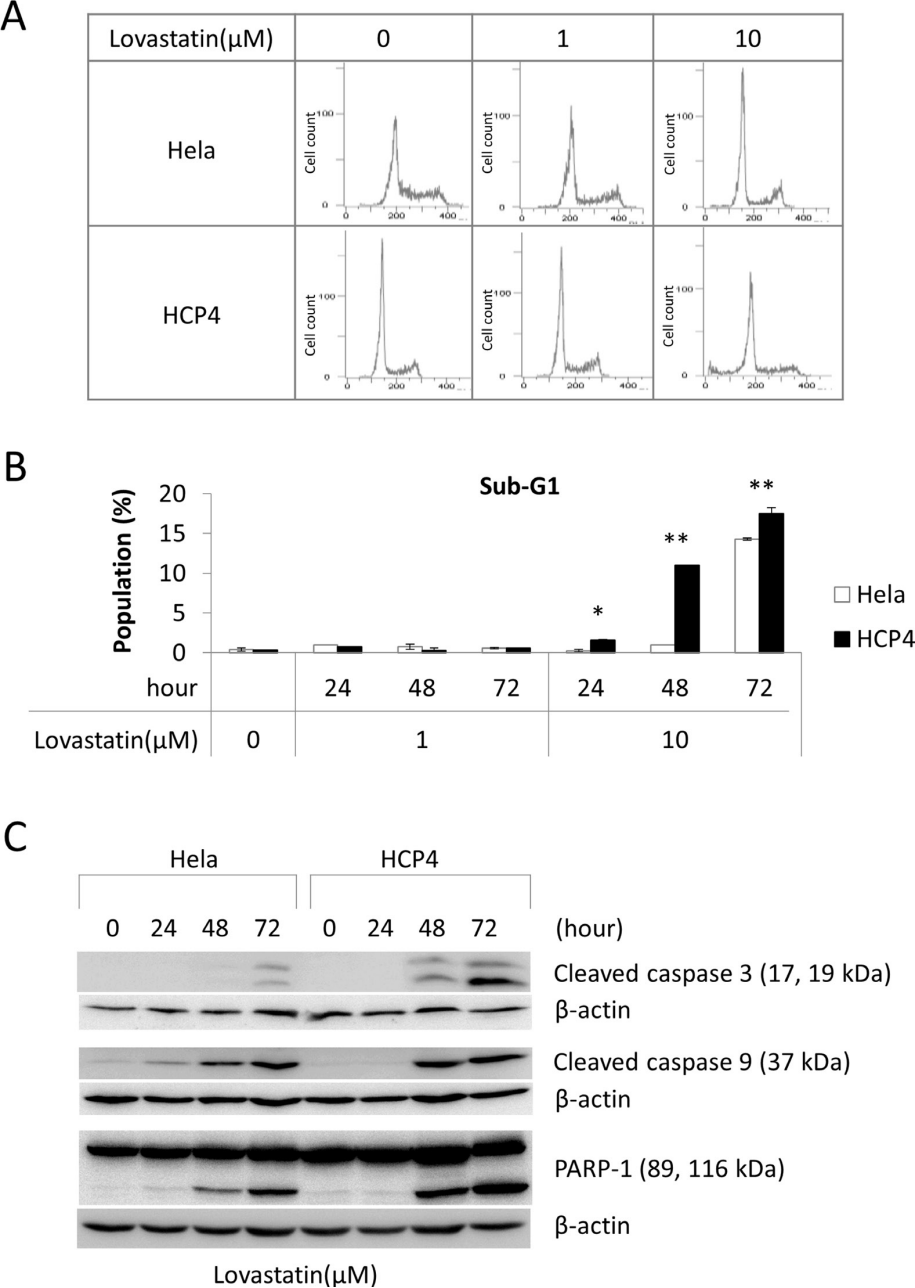
Lovastatin induced apoptosis of Cisplatin-resistant cells (**A**) Hela and HCP4 cells were treated with the indicated concentration of Lovastatin for 48 h. Cell cycle analysis was performed by flow cytometry. (**B**) The sub-G1 population was calculated form the results of (A). All values represent the mean of at least three independent experiments. Significant differences are compared for each cell line under the same condition; * and ** indicate *P* < 0.05 and *P* < 0.01, respectively. (**C**) Hela and HCP4 cells were treated with 1 µM Lovastatin for the indicated time. Lysates (50 µg) were subjected to western blot analysis with the indicated antibodies.

To determine the mechanism of viability reduction of Lovastatin for HCP4 cells we performed cell cycle analysis after treatment with Lovastatin. We used Lovastatin at 1 µM and 10 µM concentrations because 10 µM Lovastatin strongly reduce the viability of both Hela and HCP4 cells (by approximately 70% and 5%, respectively), while 1 µM Lovastatin could reduce the viability of HCP4 cells (by approximately 50%) but could not of Hela cells (Figure 1). As shown in Figure 2A and 2B, 10 µM Lovastatin significantly increased the sub-G1 population of HCP4 cells, but not Hela cells, after 48 h. These results suggested that 10 µM Lovastatin preferencally induced apoptosis of HCP4 cells. Next, we performed western blot analysis to examine the expression of the apoptosis-related proteins cleaved caspase 3, cleaved caspase 9, and PARP-1. As shown in Figure 2C, cleaved caspase 3, 9 and PARP-1 proteins were strongly induced by Lovastatin in a time-dependent manner in HCP4 cells. These proteins were also induced by Lovastatin in Hela cells, but the expression levels were low compared with HCP4 cells. These results indicated that Lovastatin induced apoptosis in Cisplatin-resistant HCP4 cells more rapidly and to a greater extent than in the parental Hela cells.

### HMGCS1 and HMGCR were upregulated in Cisplatin-resistant HCP4 cells

**Figure 3:**
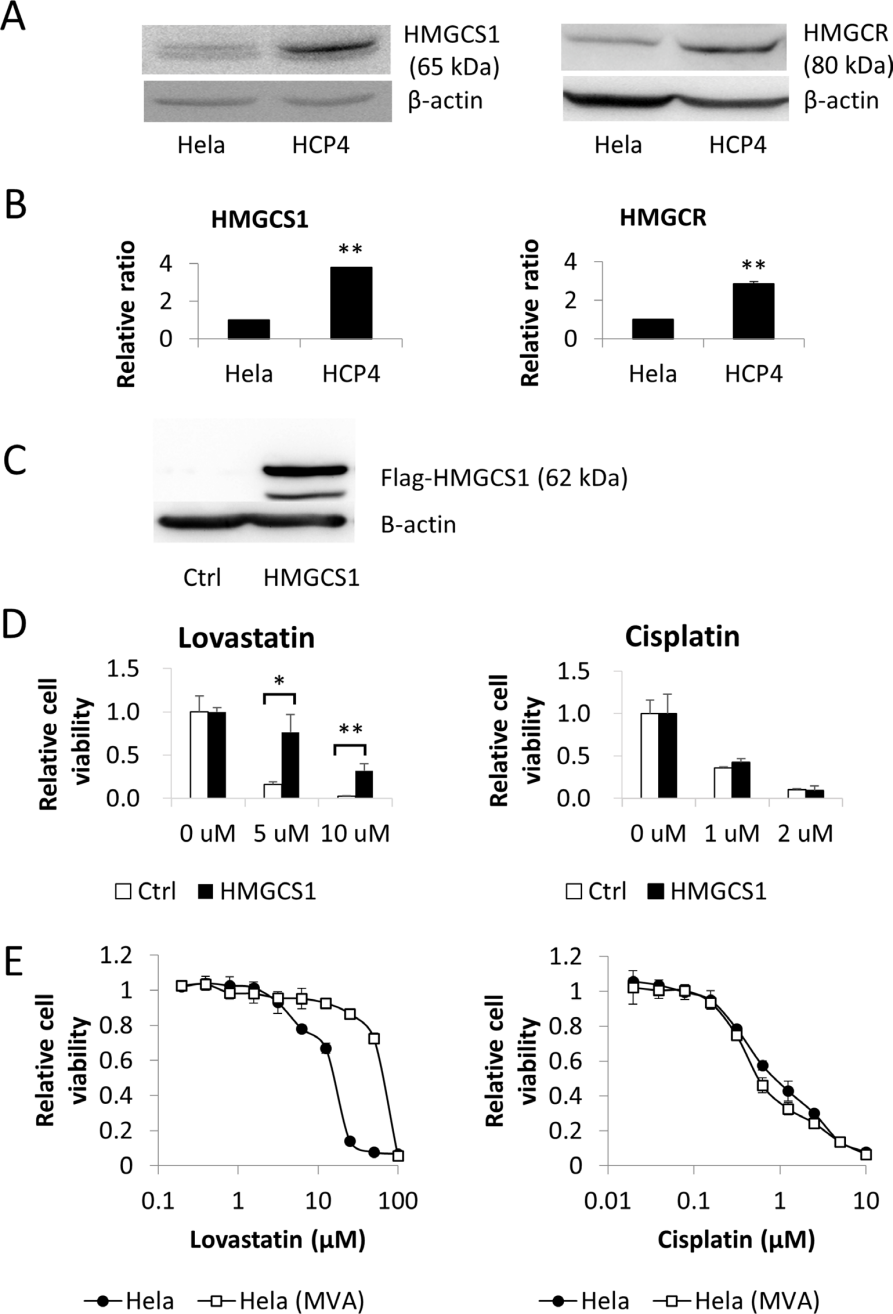
HMGCS1 was upregulated in Cisplatin-resistant HCP4 cells (**A**) Lysates (50 µg) of Hela and HCP4 cells were subjected to western blot analysis with the indicated antibodies. (**B**) Total RNA from each cell line was used for quantitative real-time RT-PCR. All values represent the mean of at least two independent experiments. mRNA expression of Hela cells was set to 1; * and ** indicate *P* < 0.05 and *P* < 0.01, respectively. (**C**) Flag-HMGCS1 and empty vector (Ctrl) were transfected into Hela cells and transfectants were selected with 3 ng/mL Puromycin for 2 weeks. Lysates (50 µg) were subjected to western blot analysis with the indicated antibodies. (**D**) Transfectants were treated with the indicated concentration of Lovastatin or Cisplatin. After 72 h, cells expressing GFP were quantified with a LUNA-FL™ Dual Fluorescence Cell Counter. All values represent the mean of at least three independent experiments. * and ** indicate *P* < 0.05 and *P* < 0.01, respectively. (**E**) Hela(MVA) indicates Hela cells cultured with 100 µM MVA for 1 month. Hela and Hela(MVA) cells were treated with serial dilutions of Lovastatin (left panel) or Cisplatin (right panel). After 72 h, the surviving cells were stained with TetraColor ONE for 2–3 h. MVA (100 µM ) was added to Hela(MVA) cells until the end of the assay. All values represent the mean of at least two independent experiments.

To clarify the mechanism underlying the sensitivity of Cisplatin-resistant HCP4 cells to Lovastatin we examined the MVA cascade. Western blot analysis revealed that cellular expression levels of HMGCS1 and HMGCR in HCP4 cells were 2.6-fold and 2.9-fold higher than those in Hela cells, respectively (Figure 3A). Real-time PCR analysis showed that the mRNAs of these genes were also upregulated in HCP4 cells (Figure 3B). Next, we performed metabolome analysis for Hela and HCP4 cells and found that the ratio of the amount of HMG-CoA in Hela cells to HCP4 cells was 1.1 (data not shown). These results suggested that the MVA cascade was activated in HCP4 cells compared with Hela cells, but the metabolized HMG-CoA was not accumulated in HCP4 cells.

### Mevalonic acid cascade is not involved in Cisplatin resistance

As enhancement of the MVA cascade in Cisplatin-resistant cells was suspected we examined the effect of MVA on Cisplatin sensitivity. First, we performed a cell viability assay with Hela cells overexpressing HMGCS1 and GFP independently (Figure 3C and Supplementary Figure 1). These cells were treated with Cisplatin or Lovastatin at the indicated concentration and GFP-expressing cells were counted. As shown in Figure 3D, there was a marked difference in sensitivity to Lovastatin between HMGCS1 overexpressing Hela cells and control cells but not to Cisplatin. Furthermore, to elucidate whether MVA was involved in Cisplatin resistance, we added Cisplatin to Hela cells that had been cultured for 1 month with 100 µM MVA. As shown in Figure 3E, IC_50_ ratios of Hela(MVA) for Hela to Lovastatin and Cisplatin were 48.8 and 0.9, respectively. These data indicate that administration of MVA to HeLa cells confers resistance to Lovastatin but not to Cisplatin.

### Lovastatin induced *KLF2*, *KLF6*, and *RHOB* expression

**Figure 4:**
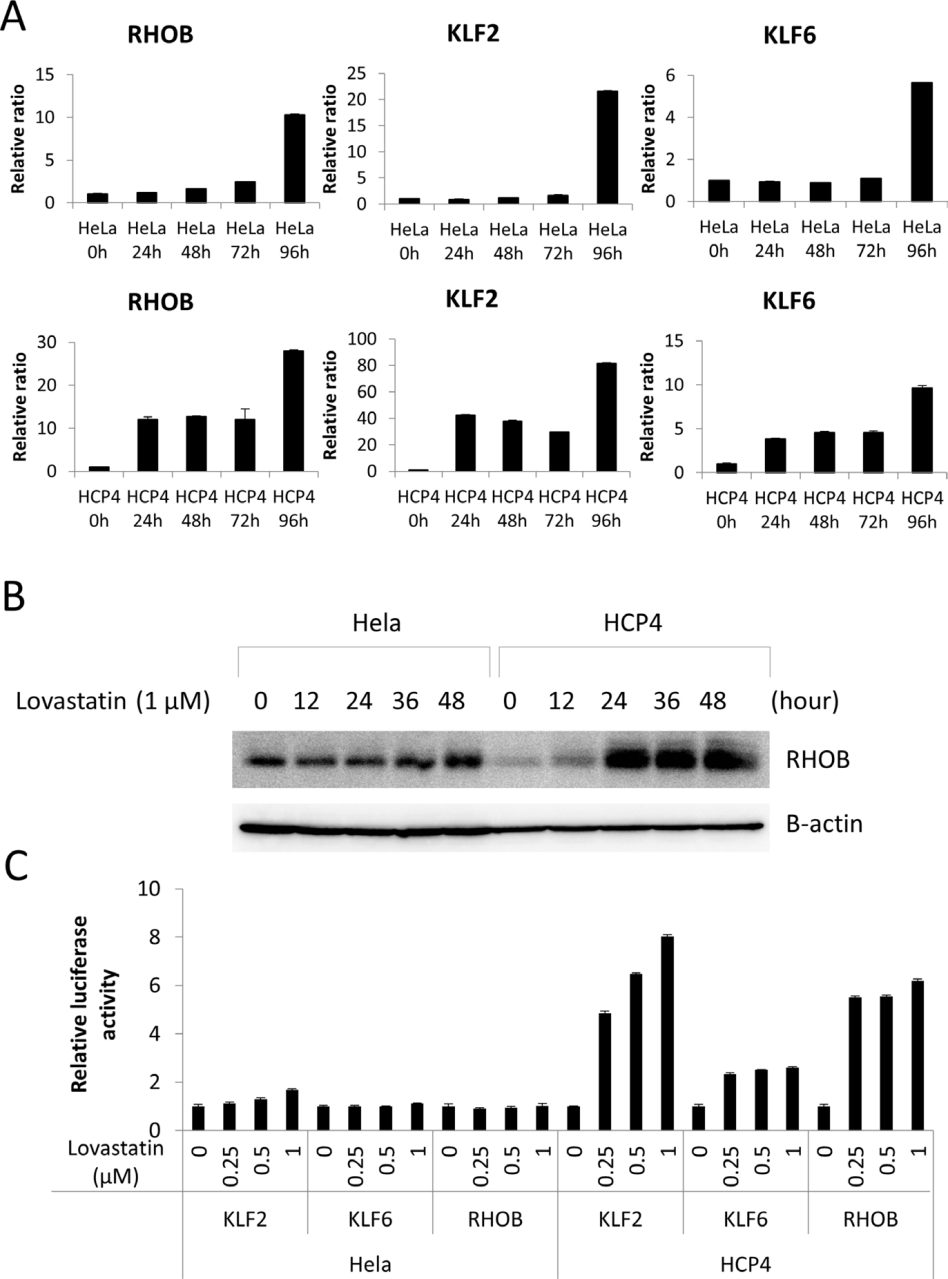
Lovastatin induced *KLF2*, *KLF6*, and *RHOB* expression (**A**) Hela and HCP4 cells were treated with 1 µM Lovastatin for indicated time. Total RNA of each cell was used for quantitative real-time RT-PCR. mRNA expression of untreated Hela and HCP4 cells was set to 1. All values represent the mean of at least two independent experiments. (**B**) Hela and HCP4 cells were treated with 1 µM Lovastatin and collected at indicated time. Lysates (25 µg) were subjected to western blot analysis with the anti-*RHOB* antibody. (**C**) Hela and HCP4 cells with integration of KLF2, KLF6, or *RHOB* promoter–luciferase gene were treated with 0, 0.25, 0.5 1 µM Lovastatin. After 48 h, a luciferase assay was carried out. The results were normalized to protein concentration and are representative of at least three independent experiments.

To elucidate the mechanism by which Lovastatin sensitized the Cisplatin-resistant cells we performed mRNA microarray analysis of HCP4 and PCDP5 cells treated with or without 1 µM Lovastatin. We found that 65 mRNAs and 54 mRNAs were induced more than 2-fold by Lovastatin treatment, HCP4 (Supplementary Table 2) and PCDP5 (Supplementary Table 3) cells, respectively. Only three genes, *KLF2*, *KLF6*, and *RHOB,* were commonly increased more than 2-fold. Expression levels of these mRNAs were verified by real-time PCR. As shown in Figure 4A, *KLF2*, *KLF6*, and *RHOB* mRNA levels were elevated in HCP4 cells after 24 h treatment with 1 µM Lovastatin. However, in HeLa cells, strongly elevated levels of these mRNAs was only observed after 96 h treatment with 1 µM Lovastatin. Another statin, Compactin, also induced expression of *KLF2* and *RHOB* mRNAs, but not *KLF6* mRNA (Supplementary Figure 2). Next, cellular protein levels of *RHOB* were investigated. As shown in Figure 4B, in the normal culture condition, *RHOB* protein of Hela cells was higher than that of HCP4 cells. However, Lovastatin induced rapidly and strongly *RHOB* protein expression in HCP4 cells (Figure 4B). Unfortunately, some antibodies against *KLF2* and *KLF6* were purchased, but specificities could not be confirmed. To elucidate whether Lovastatin induced the promoter activities of these genes, Hela and HCP4 cells stably transfected with promoter-luciferase constructs were treated with Lovastatin. As shown in Figure 4C, promoter activity of three genes were induced by Lovastatin and these inducible promoter activities were correlated with mRNA expressions levels.

### *KLF2, KLF6*, and *RHOB* led to viability reduction

**Figure 5:**
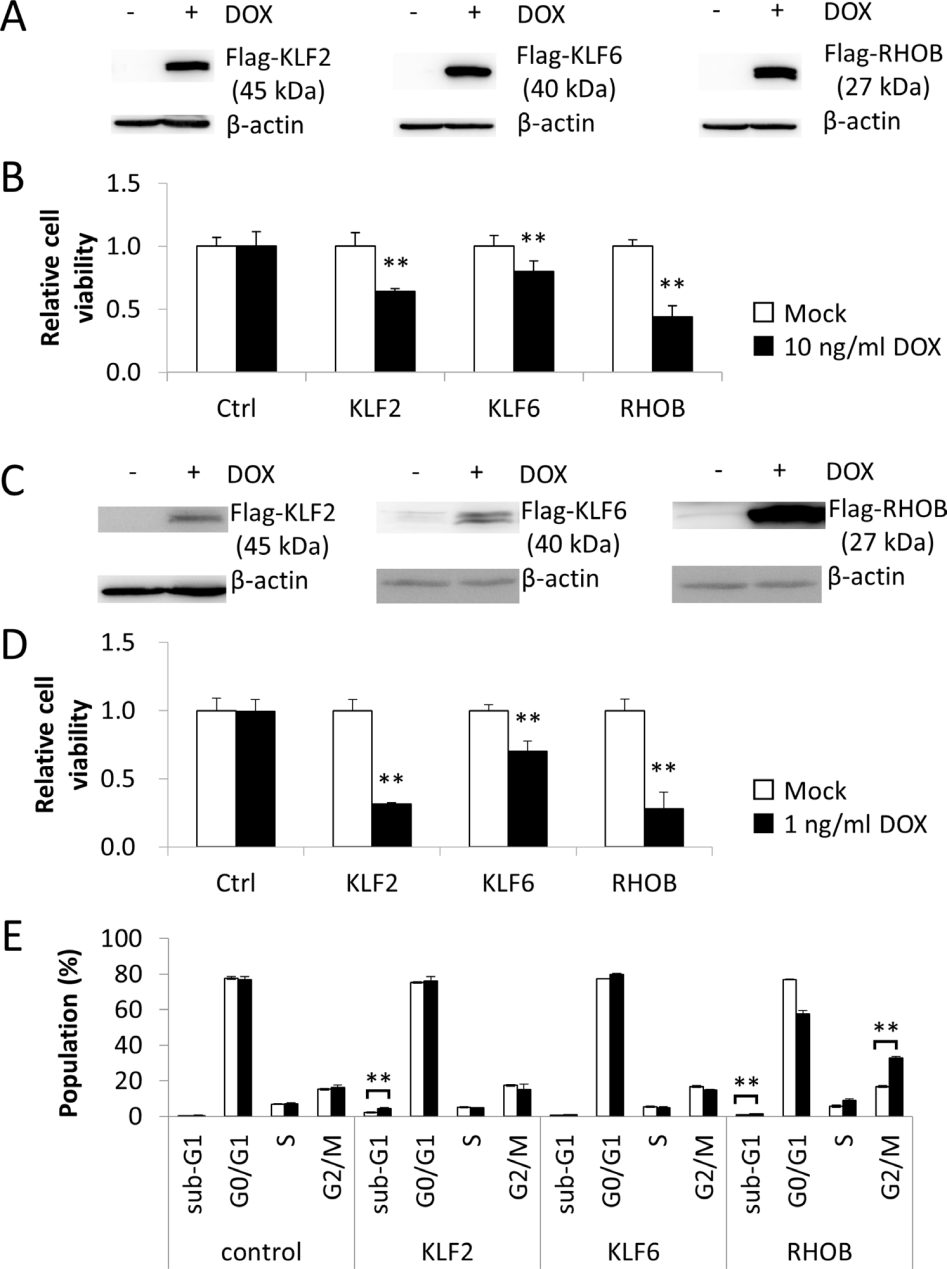
*KLF2*, *KLF6*, and *RHOB* promoted viability reduction (**A**) Hela cells were transfected with plasmids containing DOX -inducible Flag-KLF2, Flag-KLF6 and Flag-*RHOB* genes and transfectants were selected with 3 ng/mL Puromycin for 2 weeks. Transfectants were treated with 10 g/mL DOX for 24 h and cell lysates (50 µg) were subjected to western blot analysis with anti-Flag antibody. (**B**) Transfectants were treated with 10 g/mL DOX for 48 h and cells expressing GFP were quantified with a LUNA-FL™ Dual Fluorescence Cell Counter. All values represent the mean of at least three independent experiments. * and ** indicate *P* < 0.05 and *P* < 0.01, respectively. (**C**) and (**D**) The same analysis of (A) and (B) were carried out using COS-1 cells. * and ** indicate *P* < 0.05 and *P* < 0.01, respectively. (**E**) The populations of sub-G1, G0/G1, S and G2M were calculated form the results of flow cytometer. All values represent the mean of at least three independent experiments.

To clarify the mechanism of *KLF2*, *KLF6*, and *RHOB* expressions induced by Lovastatin, we used inducible expression plasmids of the Tet-On system (Supplementary Figure 3). These plasmids contained Flag-tagged *KLF2*, *KLF6*, and *RHOB* driven by the tetracycline response element together with expression sets of rtTA, GFP, and a Puromycin resistance gene (Supplementary Figure 3). Transiently transfected Hela cells and COS1 cells were cultured with 3 ng/mL Puromycin for 2 weeks and selected transfectants were treated with DOX to induce expression of *KLF2*, *KLF6*, and *RHOB*. DOX induced expression of Flag-*KLF2*, Flag-*KLF6*, and Flag- *RHOB* in Hela cells after 24 h. (Figure 5A). At 72 h after DOX treatment, GFP-expressing cells were counted. The number of cells expressing *KLF2*, *KLF6*, or *RHOB* was respectively decreased after DOX treatment, but DOX did not reduce the viability of control cells (Figure 5B). DOX also decreased the GFP-expressing COS1 cells as correlated with induced expression of *KLF2*, *KLF6*, or *RHOB* (Figure 5C and 5D). Cell cycle analysis revealed that *KLF2* and *RHOB* significantly induced an increase in the populations of sub-G1 and G2/M cells, respectively (Figure 5E).

### Effect of combination treatment with Lovastatin and Cisplatin on viability reduction

We investigated *in vitro* combination treatment of cell lines with Cisplatin and Lovastatin. The combination ratio of Cisplatin vs Lovastatin was determined by using a concentration that had relative cell viability, between 0.2 and 0.8 (Table 3). The relative cell viabilities of 2 µM Cisplatin and 10 µM Lovastatin for Hela cells were 2.4 and 0.6, respectively. Therefore, the combination ratio of Cisplatin and Lovastatin used was 2 µM: 10 µM (=1:5). Other combination ratios were determined using the same method. According to the protocol of CalcuSyn software, each single and combination treatments were performed, and the combination index (CI) of ED50, ED75, and ED90 for Hela, HCP4, PC3 and PCDP5 were calculated (Table 3). These results indicated that Lovastatin had an agonistic effect on the viability reduction of Cisplatin in Cisplatin-sensitive Hela and PC3 cells and an antagonistic effect in Cisplatin-resistant HCP4 and PCDP5 cells.

**Table 3:** Combination Index

	Combination ratio ( Cisplatin : Lovastatin )	Combination index (CI)		
		ED50	ED75	ED90
Hela	1 : 5	0.602	0.495	0.417
HCP4	10 : 1	1.437	1.812	2.412
PC3	1 : 5	0.698	0.697	0.608
PCDP5	20 : 1	1.084	1.760	3.057

## DISCUSSION

Statins are well-known inhibitors of HMGCR and are used clinically for the treatment of hyperlipidemia [5]. Recently, it was reported that statins have antitumor activity in various tumor cells, including breast, cervical, colon, endometrial, lung, pancreatic, prostate, and ovarian cancer cells, *in vitro* and *in vivo* [6, 7]. Clinical studies also indicated that the antitumor activity of statins was caused by the pleiotropic effects of statins including cell cycle arrest, induction of apoptosis, reduction of metastatic potential, inhibition of angiogenesis, and differentiation of tumors [6, 7, 21].

In this study, we investigated the antitumor effect of statins in Hela and PC3 cells as well as HCP4 and PCDP5 cells, Cisplatin-resistant cell lines established from each parent cells. Lovastatin and other statins had an antitumor effect in Hela and PC3 cells as expected. Interestingly, these statins had a strong antitumor effect in Cisplatin-resistant cells compared with each parental cell. We also investigated Lovastatin sensitivity using Cisplatin-resistant, Oxaliplatin-resistant, and Mithramycin-resistant cell lines derived from parental T24 cells. Oxaliplatin is a third-generation Cisplatin [22] and Mithramycin is a G–C-specific DNA binding antibiotic that inhibits RNA synthesis [23]. Lovastatin sensitized platinum-resistant cells but had no such sensitizing effect on Mithramycin-resistant cells. Jang *et al.* have reported that Irinotecan-resistant human colorectal adenocarcinoma HT-29 cells are approximately 2-fold more resistant to Simvastatin, when compared with parental cells [24]. These results suggest that the sensitizing effects of statins on cell viability may be specific for platinum-resistant cells. To evaluate this specificity, it is necessary to investigate the effect of Lovastatin using cells that have acquired resistance to platinum-based therapies through a variety of mechanisms. Lovastatin increased sub-G1 population and activated caspase cascade, these suggesting that apoptosis was involved in the viability reduction of Lovastatin. In general, agents that overcome anticancer drug resistance target drug resistance–related genes, and such agents can be used to sensitize drug-resistant cancers with the anticancer drug [25]. The most important result of this study is that statins alone sensitized Cisplatin-resistant cells. Furthermore, combination therapy with Cisplatin and Lovastatin had an agonistic effect on Cisplatin-sensitive cells, but an antagonistic effect on Cisplatin-resistant cells. This is the first report indicating that statins may have the potential to overcome Cisplatin resistance as single-agent therapy.

The MVA pathway is a metabolic pathway that uses acetyl-CoA to produce sterols and isoprenoids [5]. These products are essential for tumor growth and progression [8, 9, 26]. Several reports have demonstrated that statins, a class of HMGCR inhibitors, decreased the proliferation and induced apoptosis of cancer cells [27–30]. However, the association between the MVA pathway and drug-sensitive/resistance is not known. In this study we investigated whether the MVA pathway is involved in Cisplatin resistance. Protein and mRNA expression of HMGCS1 and HMGCR was higher in Cisplatin-resistant cells than in the parental cells, but the amount of HMG-CoA was almost the same between these cell lines. These data indicate that HMG-CoA synthesized by HMGCS1 might be is rapidly converted to MVA by HMGCR in Cisplatin-resistant cells. Cells overexpressing HMGCS1 were able to acquire resistance to Lovastatin but not Cisplatin. Furthermore, administration of MVA suppressed the effects of Lovastatin on cell viability but had no effect on Cisplatin sensitivity. These results indicated that the MVA pathway might not be involved in Cisplatin resistance.

Lovastatin preferentially induced the apoptosis of Cisplatin-resistant cells compared with Cisplatin-sensitive cells. To elucidate the mechanism of apoptosis induction by Lovastatin we performed cDNA microarray analysis with two types of Cisplatin-resistant cells and found that only three genes, *KLF2*, *KLF6*, and *RHOB*, were commonly upregulated over 2-fold by treatment with Lovastatin. Several previous studies demonstrated that statins increased *KLF2* and *RHOB* [31–35] and that these genes are associated with apoptosis or the cell cycle [13, 20, 36]. *KLF2* and *KLF6* belong to the family of Kruppel-like zinc finger transcription factors, which in humans contains at least 26 members including Sp1-like (Sp1-9) and KLF-like factors (KLF1-17) [37, 38] that regulate remarkably diverse processes including cell growth, signal transduction, and differentiation 10 11. *RHOB* has been reported to be downregulated in various tumors including gastric, lung, and ovarian cancer, and its overexpression inhibits proliferation, migration, and invasion [39–41]. We found that Lovastatin induced accumulation of these mRNAs by transcriptional regulation. The protein expression of *RHOB* in Cisplatin-resistant HCP4 cells was lower than that in Hela cells. Of note, *RHOB* protein in HCP4 cells was rapidly and strongly induced by Lovastatin. These results suggested that Lovastatin significantly induced *KLF2*, *KLF6* and *RHOB* expression in Cisplatin-resistant cells by transcriptional regulation. It is necessary to clarify the mechanism about transcriptional regulation by Lovastatin in Cisplatin-resistant cells including signal transduction.

Even if individual treatment with an anticancer agent reduces the viability of cancer cells, this viability reduction might be antagonized when the agents are used in combination. Recently, we reported that the aurora kinase B inhibitor, AZD1152-hQPA, had an antagonistic effect on the viability reduction of Cisplatin [42]. Both AZD1152-hQPA [43] and Cisplatin [44] induced G2/M arrest. We speculate that the use of combined drugs that promote G2/M arrest may be involved in viability reduction antagonism. In this study, we did not observe the induction of G2/M arrest by Lovastatin. However, Lovastatin increased *RHOB* expression and increased the *RHOB*-induced G2/M arrest. Jang *et al.* have reported that Simvastatin and Irinotecan have a synergistic effect on Irinotecan-resistant HT-29 cells [24]. Like Cisplatin, Irinotecan has been reported to induce G2/M arrest [44, 45]. However, Simvastatin has contrasting effects on Cisplatin-resistant and Irinotecan-resistant cells. The underlying mechanism of acquired resistance for each drug may explain these differences. In this study, the MVA pathway was not involved in Cisplatin resistance, and so it is necessary to investigate whether this pathway has a role in Irinotecan resistance. There is a possibility of exacerbating cancer, if statins are used with Cisplatin without knowing that cancer cells are resistant to Cisplatin. In short, combination therapy with Cisplatin and statins should be performed with caution in patients who relapse after using Cisplatin. In the future, it is necessary to clarify the mechanism about antagonism in combination of Cisplatin and statins.

We have identified a new activity of statins that may have the potential to overcome Cisplatin resistance as single-agent therapy. This raises the possibility that statins might relieve the suffering of patients with Cisplatin-resistant cancer. Regarding the mechanism of viability reduction induced by statins, we found the tumor suppressor genes *KLF2*, *KLF6*, and *RHOB* were rapidly and strongly accumulated in Cisplatin-resistant cells compared with Cisplatin-sensitive cells; however, the underlying mechanism remains unknown. Elucidation of this mechanism will contribute to the discovery of new target genes and the development of molecular targeted agents.

## MATERIALS AND METHODS

### Cell culture

Human prostate cancer PC3 cells and Cisplatin-resistant PCDP5 cells, human cervical cancer Hela cells and Cisplatin-resistant HCP4 cells were previously described [46, 47]. DDP10, OX2 and MM4 cells were established with human bladder cancer T24 cells to be resistant to Cisplatin, Oxaliplatin and Mithramycin, respectively. DDP10 cells and OX2 cells were previously described [48, 49]. Mithramycin-resistant MM4 cells were established from T24 cells with increasing concentrations of Mithramycin step by step. All cells were cultured in RPMI 1640 medium with GlutaMAX™ supplement (Invitrogen; Thermo Fisher Scientific, Inc., Waltham, MA, USA) containing 10% fetal bovine serum (HyClone; GE Healthcare Life Sciences, Logan, UT, USA). Cisplatin-resistant HCP4, PCDP5 and DDP10, Oxaliplatin-resistant OX2 and Mithramycin-resistant MM4 cells were usually cultured with medium containing 1 µM Cisplatin, 5 µM Oxaliplatin and 1 µM Mithramycin, respectively, which was removed from the medium one week before assay. Hela(MVA) cells were cultured with 100 µM DL-mevalonolactone (sc-211365, Santa Cruz Biotechnology) for one month or over. The COS-1 fibroblast-like cell lines derived from monkey kidney tissue (kindly gifted by Professor Keiko Funa, Sahlgrenska Cancer Center at the Sahlgrenska Academy, University of Gothenburg) was grown in Dulbecco’s modified Eagle’s medium with GlutaMAX™ supplement (Invitrogen) containing 10% fetal bovine serum (HyClone). All cell lines were incubated in 5% CO_2_ at 37°C.

### Antibodies and treatment agents

Anti-HMGCS1 (sc-33829), anti-HMGCR (sc-27578), anti-*RHOB* (sc-180), and anti-PARP-1 antibodies were purchased from Santa Cruz Biotechnology (Santa Cruz, CA). Anti–cleaved caspase 3 (#9661) and anti–cleaved caspase 9 (#9501) antibodies were purchased from Cell Signaling Technology (Beverly, MA). Anti-Flag M2 (F3165) and anti–β-actin (A2228) antibodies were from Sigma-Aldrich (St. Louis, MO). Simvastatin (196-17801), Pravastatin sodium salt (162-19821), Lovastatin (125-04581), Compactin (033-17031), Fluvastatin sodium (069-05571), Atorvastatin calcium trihydrate (012-23901), and Pitavastatin calcium (163-24861) were purchased from Wako Pure Chemical Industries Ltd. (Osaka, Japan).

### Plasmid construction

To obtain cDNAs of *HMGCS1*, *KLF2*, *KLF6*, and *RHOB*, a cDNA library (Human Universal QUICK-Clone™ cDNA II, Clontech Laboratories, Inc., Palo Alto, USA) was PCR amplified using specific primer pairs (Supplementary Table 3). The PCR product of HMGCS1 cDNA containing Flag sequence at N-terminal was ligated into the MCS of pEB GFP-T2A-Puro expression plasmid (Supplementary Figure 1). Flag-tagged *KLF2*, *KLF6* and *RHOB* cDNAs were ligated into the MCS of pEB Tet-On GFP-T2A-Puromycin expression plasmid (Supplementary Figure 3). To prepare the *KLF2*, *KLF6*, and *RHOB* promoter resions, genomic DNA was amplified using specific primer pairs (Supplementary Table 4). The PCR products of promoter resions were cloned and ligated into the MCS of Lenti Luc2P GFP-T2A-Puromycin reporter plasmid (Supplementary Figure 4).

### Transfection of expression plasmids

Hela or COS1 cells were transfected with expression plasmids (Supplementary Figure 1 and 3) capable of replication in mammalian cells through EBNA1 and OriP gene expression (Wako Pure Chemical Industries, Tokyo, Japan). Transfection was performed with X-tremeGENE 9 DNA transfection reagent (Roche Life Sciences, Indianapolis, IN) according to the manufacturer’s protocol [23]. After transfection and culture in medium containing 3 ng/mL Puromycin (Invitrogen, Carlsbad, CA) for 2 weeks, more than 90% of the cells expressed GFP. Ectopic Flag-HMGCS1 was expressed by the CMV promoter, and Flag-*KLF2*, Flag-*KLF6*, and Flag-*RHOB* were expressed by the Tet-On system (Supplementary Figure 3).

### Infection of lentivirus

A Lenti Luc2P GFP-T2A-Puromycin reporter plasmid (Supplementary Figure 4) was transfected to HEK293TN cells (System Biosciences, CA, USA) with pPACKH1 Lentivector Packaging Kit (#LV500A-1; System Biosciences) according to the manufacturer’s protocol. After 12 hours, the culture medium was changed, and cells were further cultured for 24 hours. Culture medium containing lentivirus was collected and centrifuged for 5 min at 8,000 rpm. The supernatant was transferred to Hela or HCP4 cells. After 48 hours, the medium was changed to fresh medium containing 3 ng/mL Puromycin, and the cells were cultured for 2 weeks. Before the assay, we confirmed that more than 90% of the cells expressed GFP.

### Cell viability assays

Hela cells (1 × 10^3^) or HCP4 cells (2 × 10^3^) were seeded into 96-well plates for 24 h and then treated with Cisplatin or statins at the maximum concentration indicated and 2-fold serial dilutions. For combination treatment with Lovastatin and Cisplatin, the indicated fixed concentration ratios were used. After 72 h, the surviving cells were stained with cell proliferation assay kit (TetraColor ONE; Seikagaku Corporation, Tokyo, Japan) for 2–3 h at 37°C and absorbance was measured at 450 nm, according to the manufacturer’s protocol. To measure the half maximal inhibitory concentration (IC50) in each experiment, CalcuSyn software version 2.0 (Biosoft, Cambridge, UK) was used. For combination treatment of Cisplatin and Lovastatin, the combination index (CI) calculated by CalcuSyn software was employed as previously reported [42]. For analysis of transfectants, cells expressing GFP were counted using a LUNA-FL™ Dual Fluorescence Cell Counter (Logos Biosystems, Gyunggi-do, South Korea) according to the manufacturer’s protocol.

### Flow cytometry

The flow cytometric analysis has been described previously [50]. Hela and HCP4 cells (2.5 × 10^5^ cells/well) were seeded in 6-well plates and incubated overnight before treatment with 0, 1, and 10 µM Lovastatin. After 24 h, 48 h, and 72 h, cells were harvested, washed twice with ice-cold PBS, resuspended in 70% ethanol, and stored at −20°C until use. Cells transfected with Flag-*KLF2*, Flag-*KLF6*, and Flag-*RHOB* expression plasmids were treated with 1 ng/mL DOX for 48 h, washed twice with ice-cold PBS, and stained with the Muse Cell Cycle Kit (Millipore). The cells were analyzed using a flow cytometer (EC800 Analyzer, Sony Co., Ltd., Tokyo, Japan).

### Western blot analysis

Preparation of whole cell lysates and western blot analysis were performed as described previously [50]. Briefly, 50 µg of whole cell lysate was separated by 8–15% SDS-PAGE and transferred onto polyvinylidene difluoride membranes. The bound antibody was visualized using an enhanced chemiluminescence kit (GE Healthcare Bio-Sciences, Pittsburgh, PA, USA) and the signal intensity was quantitated using Multi Gauge software version 3.0 (Fujifilm, Tokyo, Japan).

### RNA extraction, reverse transcription, and quantitative real-time PCR analysis

Total RNA of Hela and HCP4 cells under the indicated conditions was extracted by RNeasy Mini Kit (Qiagen, Valencia, CA, USA) according to the manufacturer’s protocol and used for real-time PCR or mRNA microarray analysis as described previously [50]. Briefly, quantitative real-time RT-PCR analysis was carried out with the appropriate primer sets (Supplementary Table 5) using the 7500 Fast Real-Time PCR System (Applied Biosystems, Foster City, CA, USA). The comparative cycle time (ΔΔCT) method was used to quantify gene expression. Values were normalized to those for human β-actin. All samples were run in duplicate in each experiment.

### Microarray analysis

Total RNA of HCP4 cells with or without Lovastatin treatment was extracted by miRNeasy Mini Kit (Qiagen) and 1 µg or 0.25 µg of total RNAs were used for 3D-Gene mRNA. Microarray analysis was performed using the 3D-Gene mRNA microarray platforms (TORAY, Kamakura, Japan). Briefly, for 3D-Gene mRNA microarray analysis, total RNA was transcriptionally amplified once using Arcturus^®^ Paradise^®^ PLUS 2 Round Kit–Amino Allyl (Life Technologies Corporation, Carlsbad, CA USA) according to the manufacturerʼs protocol. Obtained amino-allyl labeled antisense RNA (10 µg of aRNA) was labeled with Cy5-dye (GE Healthcare, Buckinghamshire, England) according to the manufacturerʼs protocol. The Cy5-labeled aRNA pools and hybridization buffer, and hybridized for 16 h. The hybridization was performed using the supplierʼs protocols (www.3d-gene.com). Detected signals for each gene were normalized by global normalization method (the median of the detected signal intensity was adjusted to 25.

### Metabolic assay

Hela cells (3.47 × 10^3^) or HCP4 cells (6.15 × 10^3^) were plunged into 2 mL of 5% Mannitol/Milli-Q water containing internal standards (Solution ID: 304-1002, Human Metabolome Technologies, Inc., Tsuruoka, Japan) at 0°C to inactivate enzymes. Sample preparation and metabolome measurements by CE-MS analysis were carried out through a facility service at Human Metabolome Technologies Inc., Tsuruoka, Japan [51].

### Reporter assays

Hela or HCP4 cells were infected with lentivirus including *KLF2*-, *KLF6*-, *RHOB*-promoter-Luc2P constructs as described above. Each cell type (1 × 10^5^/well) was seeded in a 12-well plate. After 24 hours, cells were treated with the indicated concentration of statins and cultured further for 24 hours. Luciferase activity was detected using a Picagene kit (Toyoinki, Tokyo, Japan) and measured with a luminometer (Luminescencer JNII RAB-2300; ATTO, Tokyo, Japan). Results were normalized to the protein concentration determined by the Bradford method and are representative of at least three independent experiments.

### Statistical assays

Results were compared by Student’s *t*-test and data were expressed as mean ± S.D. Statistical significance was defined as *P* < 0.05.

## SUPPLEMENTARY MATERIALS FIGURES AND TABLES







## Abbreviations

aRNA: antisense RNA
AZD1152-hQPA: AZD1152-hydroxyquinazoline pyrazol anilide
CMV: cytomegalovirus
COS1: fibroblast-like cell lines derived from monkey kidney tissue
DDP10: Cisplatin-resistant T24 cells
DOX: doxycycline
EBNA1: Epstein-Barr nuclear antigen 1
GFP: green fluorescence protein
HCP4: Cisplatin-resistant Hela cells
Hela: human cervical cancer cells
Hela(MVA): Hela cells cultured in the presence of mevalonic acid
HMG-CoA: 3-hydroxy-3-methylglutaryl-coenzyme A
HMGCR: 3-hydroxy-3-methylglutaryl-coenzyme A reductase
HMGCS1: 3-hydroxy-3-methylglutaryl-coenzyme A synthase 1
IC50: inhibitory concentration of 50%
*KLF2*: Krüppel-like factor 2
*KLF6*: Krüppel-like factor 6
KLFs: Krüppel-like factors
Luc2P: destabilized and optimized firefly luciferase with the -Pro-Glu-Ser-Thr
MCS: multicloning sites
MM4: Mithramycin-resistant T24 cells
MVA: mevalonic acid
OriP: origin of viral replication
OX2: Oxaliplatin-resistant T24 cells
PARP-1: poly (adenosine diphosphate ribose) polymerase 1
PC3: human prostate cancer cells
PCDP5: Cisplatin-resistant PC3 cells
*RHOB*: ras homolog family member B
RT-PCR: reverse transcription-polymerase chain reaction
rtTA: teteracycline (Tc)-dependent and -inducible transcriptional activator
shRNA: short hairpin ribonucleic acid
T24: human bladder cancer cells
T2A: Thosea asigna virus 2A
TRE: tetracycline response element
