# Supplementary material for: Lovastatin induced Kruppel like factor 2 (KLF2), Kruppel like factor 6 (KLF6) and Ras homolog family member B (RHOB) genes and preferentially led to viability reduction of Cisplatin-resistant cells
Source: Oncotarget. 2017 Nov 16;8(63):106429–42. doi: 10.18632/oncotarget.22472 (PMC5739745; doi:10.18632/oncotarget.22472)
Supplement: Supplementary file 1 [file oncotarget-08-106429-s001.pdf]

Lovastatin induced Kruppel like factor 2 (*KLF2*), Kruppel like factor 6 (*KLF6*) and Ras homolog family member B (*RHOB*) genes and preferentially led to viability reduction of Cisplatin-resistant cells

SUPPLEMENTARY MATERIALS

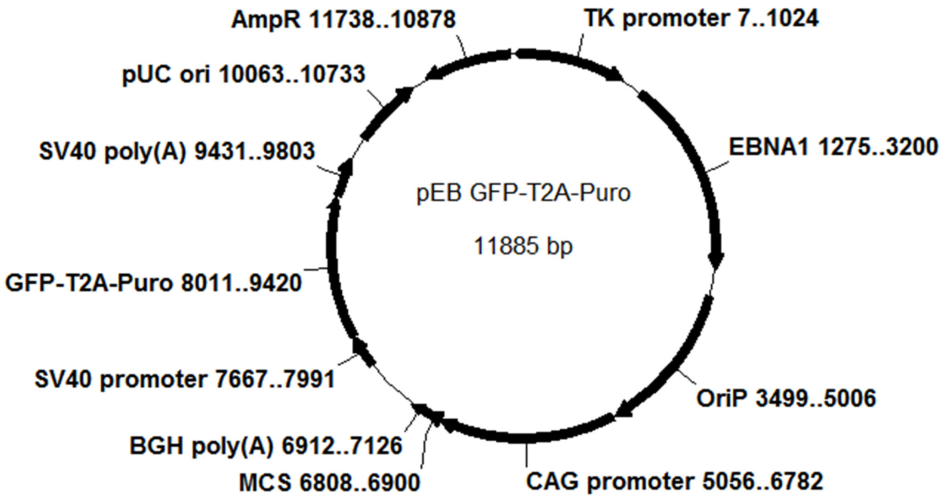

Supplementary Figure 1: pEB GFP-T2A-Puro plasmid contains EBNA1, OriP and CAG promoter sequence of pEBMulti-Neo (Wako Chemical Co., Tokyo, Japan), and GFP-T2A-Puro sequence of pCDH-EF1-MCS-BGH-PGK-GFP-T2A-Puro (System Biosciences, CA, USA). Flag tagged HMGCS1 cDNA was ligated to the MCS of this plasmid.

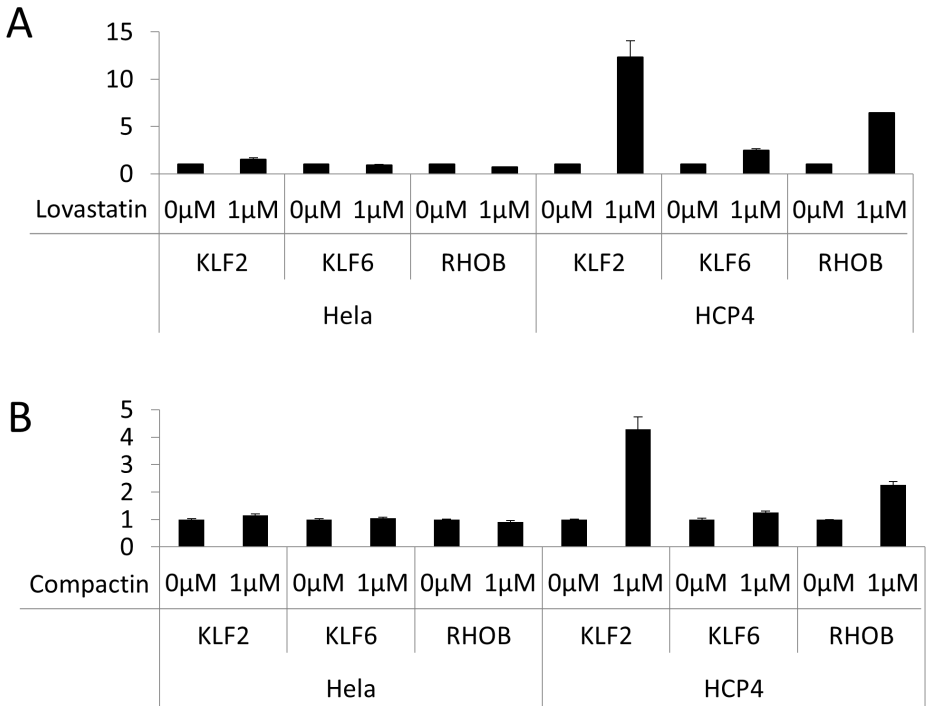

Supplementary Figure 2: Lovastatin and Compactin induced *KLF2*, *KLF6* and *RHOB* expression. Hela and HCP4 cells were treated with 1 μM Lovastatin (**A**) and Compactin (**B**) for 24 h. Total RNA of each cell was used for quantitative real-time RT-PCR. mRNA expression of untreated Hela and HCP4 cells was set to 1. All Values represent the mean of at least two independent experiments.

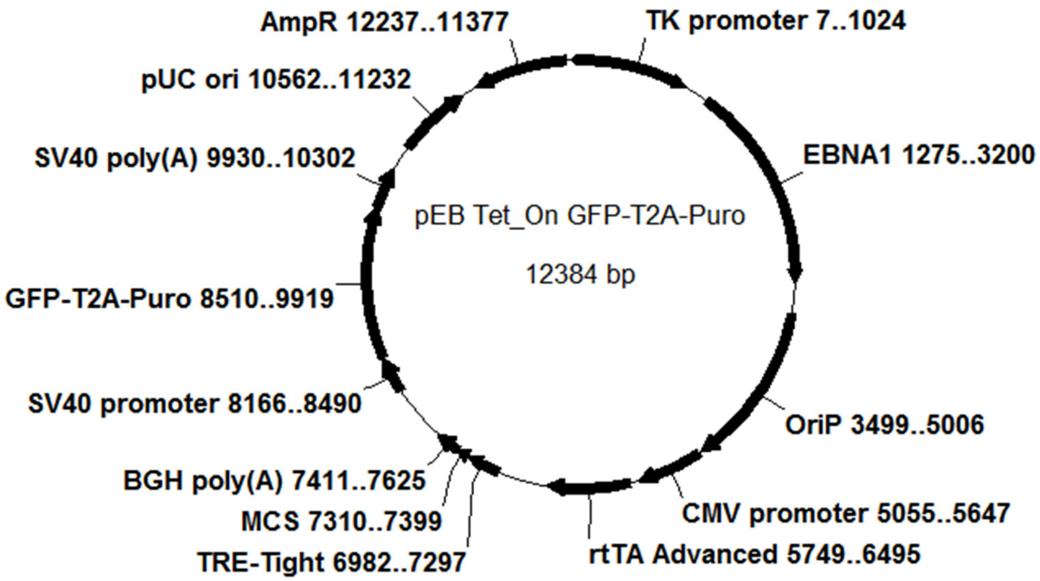

Supplementary Figure 3: pEB Tet-On GFP-T2A-Puro plasmid contains EBNA1 and OriP sequence of pEBMulti-Neo (Wako Chemical Co., Tokyo, Japan), CMV-rtTA sequence of pTet-On Advanced Vector (Clontech Laboratories, Inc., Palo Alto, CA), TRE promoter sequence of pTRE-Tight Vector (Clontech Laboratories, Inc.), and GFP-T2A-Puro sequence of pCDH-EF1-MCS-BGH-PGK-GFP-T2A-Puro (System Biosciences). Flag tagged *KLF2*, *FLF6* and *RHOB* cDNAs were ligated to the MCS of this plasmid. These proteins are induced by administration of doxycycline (Dox).

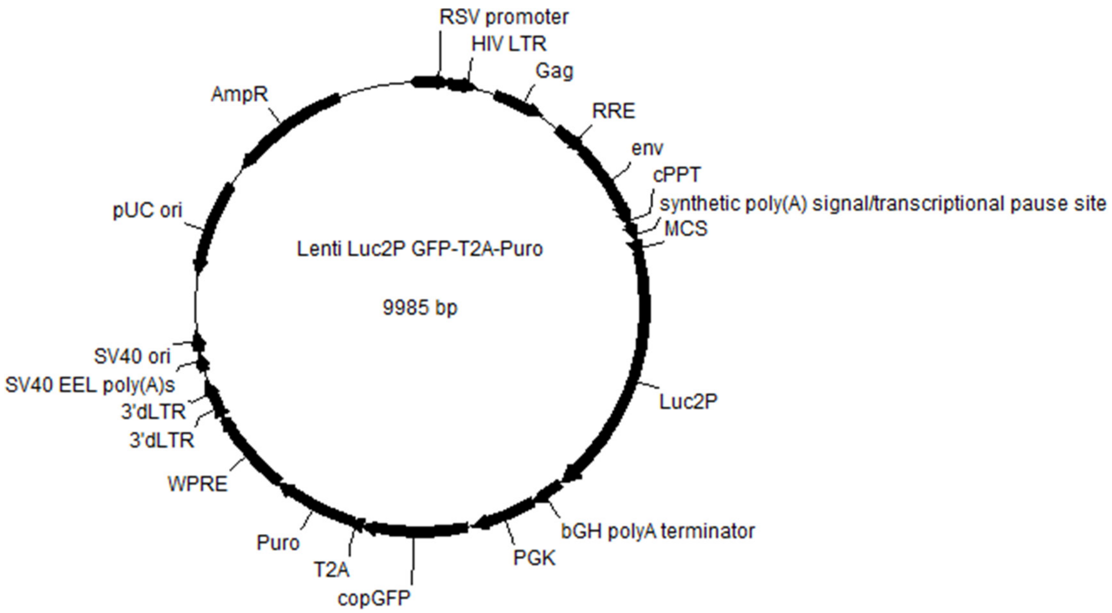

Supplementary Figure 4: Lenti luc2P GFP-T2A-Puro plasmid was constructed as follow. EF1 promoter sequence of pCDH-EF1-MCS-BGH-PGK-GFP-T2A-Puro (System Biosciences) was changed to MCS-luc2P sequence of pGL4.15 [Luc2P Hygro] (Promega Biotec, Madison, WI). Promoter resions of *KLF2, KLF6* and *RHOB* were ligated to MCS of this plasmid.

Supplementary Table 1: Evaluation of IC50

	T24	DDP10	Ratio (DDP10/T24)	Ratio (T24/DDP10)
Cisplatin (μM)	0.861 ±0.001	6.099 ±0.510	7.1	
Lovastatin (μM)	6.162 ±0.515	4.915 ±0.380		1.3
	**T24**	**OX2**	**Ratio (OX2/T24)**	**Ratio (T24/OX2)**
Oxaliplatin (μM)	2.046 ±0.186	31.817 ±3.759	15.6	
Lovastatin (μM)	6.85835 ±0.134	3.1523 ±0.618		2.2
	**T24**	**MM4**	**Ratio (MM4/T24)**	**Ratio (T24/MM4)**
Mithramycin (μM)	0.370 ±0	>100	>270	
Lovastatin (μM)	6.081 ±0.367	6.690 ±0.031		0.9

DDP10, OX2 and MM4 cells are established from T24 cells and they are resistant to Cisplatin, Oxaliplatin and Mithramycin, respectively.

Supplementary Table 2: List of 65 genes increased over 2-fold by lovastatin in HCP4 cells. See Supplementary_Table_2.

Supplementary Table 3: List of 54 genes increased over 2-fold by lovastatin in PCDP5 cells. See Supplementary_Table_3.

Supplementary Table 4: Specific primer pairs for cDNA and promoter

Table 4A: cDNAs

Gene name	Primer sequence
HMGCS1 cDNA	5′-GCCACCATGCCTGGATCACTTCCTTTG-3′
	5´-TTAATGTTCCCCATTACTAATGACAGCTGC-3′
KLF2 cDNA	5′-ATGGCGCTGAGTGAACCCATCCTG-3
	5′-CTACATGTGCCGTTTCATGTGCAGC-3
KLF6 cDNA	5′-ATGGACGTGCTCCCCATGTGCAGC-3′
	5′-TCAGAGGTGCCTCTTCATGTGCAGGG-3
RHOB cDNA	5′-ATGGCGGCCATCCGCAAGAAGCTG-3′
	5′-TCATAGCACCTTGCAGCAGTTGATGCAGCC-3′,

Start or stop codons were underlined.

Table 4B: Promoters

Gene name	Primer sequence	Position
KLF2 promoter	5′-AGATCTGTTGGGGAGGAGGGGGTGGGTGTC-3′	–961 to +80
	5′-AAGCTTGGGACCCGGGGAGAAAGGACGCGG-3′	
KLF6 promoter	5′-AGATCTCCTTGGTGAGGGCTAAGATCGCCC-3′	–901 to +167
	5′-AAGCTTGCAAACTCCAGGCTCGCAGAGACG-3′	
RHOB promoter	5′-AGATCTAAAGAGCTGCCCCTCCCCCACAGG-3′	–429 to +339
	5′-AAGCTTGCTCGCCGCTCACTGCTCACCTCG-3′	

Restriction enzyme cleavage sites for ligation were underlined.

Table 4C: shRNAs

Gene name	Primer sequence
KLF2 shRNA1	5′-GATCCGGCACCGACGACGACCTCAACAGCTTCCTGTCAGACTG<br>TTGAGGTCGTCGTCGGTGCCTTTTTG-3′
	5′-AATTCAAAAAGGCACCGACGACGACCTCAACAGTCTGACAGGA<br>AGCTGTTGAGGTCGTCGTCGGTGCCG-3′
KLF2 shRNA2	5′-GATCCGGGTGGCCTGGTGTCTGAGCTGCCTTCCTGTCAGAGCA<br>GCTCAGACACCAGGCCACCCTTTTTG-3′
	5′-AATTCAAAAAGGGTGGCCTGGTGTCTGAGCTGCTCTGACAGGA<br>AGGCAGCTCAGACACCAGGCCACCCG-3′
RHOB shRNA1	5′-GATCCAAGACGTGCCTGCTGATCGTGTTCTTCCTGTCAGAAACA<br>CGATCAGCAGGCACGTCTTTTTTTG-3′
	5′-AATTCAAAAAAAGACGTGCCTGCTGATCGTGTTTCTGACAGGAA<br>GAACACGATCAGCAGGCACGTCTTG-3′
RHOB shRNA2	5′-GATCCCCGACATTGAGGTGGACGGCAAGCTTCCTGTCAGACTTG<br>CCGTCCACCTCAATGTCGGTTTTTG-3′
	5′-AATTCAAAAACCGACATTGAGGTGGACGGCAAGTCTGACAGGA<br>AGCTTGCCGTCCACCTCAATGTCGGG-3′

Single underline and double underline indicate sense strand and anti-sense strand, respectively.

Supplementary Table 5: Primer sets used for Real-Time PCR System (Applied Biosystems)

Gene name	Primer sets
ACTB (β-actin)	Hs01060665_g1
HMGCS1	Hs00940429_m1
HMGCR	Hs00168352_m1
KLF2	Hs00360439_g1
KLF6	Hs00810569_m1
RHOB	Hs03676562_s1
